# Neural mechanisms of uncertain decision-making in anxious adolescents: an EEG study based on the balloon analogue risk task

**DOI:** 10.3389/fpsyt.2026.1918215

**Published:** 2026-09-22

**Authors:** Cheng Zhao, Jinling Song, He Wang, Yingjie Liu, Yunen Chen

**Affiliations:** North China University of Science and Technology, Tangshan, China

**Keywords:** adolescent anxiety, balloon analogue risk task, decision-making, EEG, functional connectivity, neural oscillations

## Abstract

**Background:**

Adolescence is marked by heightened vulnerability to anxiety which can impair decision-making under uncertainty. The underlying neural oscillatory network-level mechanisms in anxious adolescents require further elucidation.

**Methods:**

We recruited 40 adolescents (20 adolescents with clinically diagnosed anxiety disorders, 20 matched controls). Participants performed a gain/loss version of the Balloon Analogue Risk Task (BART) during EEG recording. Behavioral data, time-frequency EEG power in beta (13–30 Hz) and theta (4–7 Hz) bands, and phase-based functional connectivity were analyzed.

**Results:**

Anxious adolescents exhibited significant behavioral avoidance, reflected in fewer pump attempts and lower BART scores compared to controls. EEG analysis revealed elevated beta-band power during gain decisions and enhanced beta-band occipito-temporal connectivity in the anxious group. In loss contexts, anxious adolescents showed increased theta-band parieto-temporal connectivity.

**Conclusions:**

Adolescent anxiety is associated with a generalized avoidance pattern during uncertain decision-making, characterized by distinct neural dynamics. Beta-band changes in gain contexts may reflect heightened vigilance, while theta-band connectivity in loss contexts may indicate amplified threat monitoring. These neural signatures may represent candidate neurophysiological signatures of anxiety-related alterations in decision-making under uncertainty, warranting further validation in longitudinal and predictive studies.

## 1 Introduction

Anxiety disorders are among the most prevalent mental health conditions globally, characterized by excessive fear, persistent worry, and behavioral avoidance (1). Adolescence—a critical developmental stage marked by rapid psychological, cognitive, and social changes—represents a period of heightened vulnerability to the onset and persistence of anxiety. Evidence indicates that adolescents with anxiety exhibit distinct cognitive impairments, particularly in executive functions and decision-making. In uncertain contexts, they tend to underestimate potential gains and overestimate potential losses, resulting in overly conservative or avoidant choice strategies (2, 3). These biases are thought to stem from altered prefrontal–limbic interactions, which limit the effective integration of emotional and cognitive information (4, 5). Understanding the neural mechanisms underlying these impairments may therefore help identify candidate neurophysiological signatures associated with anxiety-related decision-making, providing a basis for future research on early identification and individualized interventions.

Decision-making is fundamental to adolescents’ daily functioning and psychological development, influencing academic achievement, peer relationships, resource allocation, and long-term goal pursuit (6). Within this domain, decision-making under uncertainty is particularly critical, as adolescents frequently encounter situations that require the evaluation of potential rewards, possible losses, and associated emotional responses (7). Effective decision-making under uncertainty supports adaptive functioning and healthy development. However, adolescents with anxiety often exhibit systematic biases, such as overestimating potential threats, avoiding uncertain choices, and adopting excessively cautious strategies, which may hinder adaptive exploration and limit learning opportunities (8, 9). These impairments reflect a disruption in the integration of emotion and cognition and may contribute to the persistence and exacerbation of anxiety symptoms over time (10).

Decision-making biases are systematic deviations from rational choice, encompassing cognitive and affective distortions (11). A widely used paradigm for examining these biases is the framing effect, which captures shifts in decision tendencies under uncertainty depending on whether options are presented as gains or losses (8, 12, 13). Clinical studies indicate that individuals with anxiety exhibit heightened avoidance of uncertainty across both gain and loss frames, with particularly pronounced avoidance in loss contexts (9, 14, 15). These patterns are thought to reflect distinct emotional and cognitive mechanisms: gain frames engage reward-related evaluation, whereas loss frames activate threat-related avoidance (16). Similarly, anxious adolescents demonstrate generalized avoidance in uncertain decision-making across both contexts (9, 17, 18), suggesting difficulties in flexibly adapting strategies to contextual cues. This rigidity may contribute to maladaptive real-world choices. Given that gain–loss framing captures essential aspects of everyday evaluation under uncertainty, investigating the neural mechanisms underlying framing-related biases provides a valuable framework for understanding cognitive vulnerabilities in adolescent anxiety (19, 20).

Advances in cognitive neuroscience have made it increasingly feasible to examine how neural systems support the psychological processes involved in adolescent decision-making under uncertainty. These methodological developments have facilitated research on how anxiety biases uncertainty appraisal, heightens sensitivity to potential gains or losses, and disrupts emotion regulation, thereby providing a neurophysiologically informed framework for understanding maladaptive behavior (21). To investigate the neural mechanisms underlying anxiety’s impact on uncertain decision-making in adolescents, the present study employed electroencephalography (EEG) combined with a behavioral paradigm. EEG offers high temporal resolution, allowing the capture of rapid neural dynamics during decision-making under uncertainty (22, 23). Time–frequency analyses permit the examination of oscillatory power across distinct frequency bands over defined time windows, providing indirect insights into the neural processes underlying uncertainty evaluation, reward sensitivity, and emotion regulation (24). Among these frequency bands, β (13–30 Hz) and θ (4–7 Hz) oscillations are particularly relevant to uncertain decision-making and affective control. β activity is associated with top-down cognitive control, sustained attention, and the maintenance of task-relevant representations (25), supporting goal-directed behavior through the stabilization of cognitive states and the suppression of irrelevant information (26, 27). β oscillations also reflect inhibitory control over emotional responses, implicating prefrontal regions in affect regulation; altered β synchronization in anxiety may indicate difficulties in the executive regulation of heightened affective states (28, 29). These previous findings provide developmental and theoretical context for understanding anxiety-related alterations in cognitive control and affect regulation but were not directly assessed in the present sensor-level EEG analysis. θ oscillations, particularly frontal-midline θ activity reported in previous studies, have been considered markers of cognitive control under conflict, uncertainty, or increased working memory load (30–32) and is critically involved in monitoring action outcomes, error detection, and feedback-based learning. θ rhythms are also linked to emotional processing, with increased frontal θ associated with negative affect, heightened arousal, and engagement of limbic–prefrontal circuits (23, 24). In anxiety, atypical θ modulation during uncertainty and threat-related tasks suggests excessive sensitivity to potential losses and negative outcomes (33). Together, β and θ oscillations illustrate the interaction of cognitive control and emotional processes (34). Beyond regional oscillatory activity, interactions between distributed cortical regions may provide additional insights into uncertainty processing. Previous studies have highlighted the importance of large-scale cortical interactions during cognitive control and decision-making. In the present study, we specifically examined task-related sensor-level connectivity between occipital–temporal and parietal–temporal electrode pairs based on EEG dynamics. Parietal regions contribute to attentional allocation and outcome evaluation, whereas temporal regions support emotional and contextual processing (35). Thus, parietal–temporal interactions may facilitate the integration of cognitive and affective information during uncertain decision-making. Given the heightened threat sensitivity and negative outcome monitoring in anxiety, increased theta-band synchronization between these regions may reflect enhanced neural coordination during loss-related uncertainty processing. Investigating dynamic EEG changes in anxious adolescents is therefore crucial for understanding the neural mechanisms of decision-making under uncertainty. Although β- and θ-band oscillations were the primary frequency bands of interest in the present study, alpha-band activity was also examined as an exploratory measure. Alpha oscillations have been associated with attentional allocation and inhibitory control during cognitive processing (36). Given the limited evidence regarding anxiety-related alpha alterations during uncertain decision-making, no directional hypothesis was specified for alpha-band activity. Based on the established role of β oscillations in cognitive control and affective regulation, we hypothesized that anxious adolescents would exhibit increased β-band power during gain-related uncertain decision-making due to the greater effort required to inhibit irrelevant information and regulate affective responses.

Furthermore, recent advances in network-based approaches have emphasized the importance of examining not only local oscillatory dynamics but also large-scale neural interactions. EEG functional connectivity analysis offers a powerful method to capture such interactions by quantifying phase synchronization between brain regions, thereby revealing the coupling of reward, control, and affective networks during decision-making (37, 38). Beyond identifying static connectivity patterns, this approach enables the examination of the temporal consistency of interregional coordination across trials, providing a dynamic perspective on neural communication. For example, adolescents with higher anxiety exhibit stronger β- and θ-band synchronization between frontal and limbic regions during emotion regulation tasks (39, 40). Dynamic connectivity analyses further show that large-scale neural networks undergo reconfiguration during reward-based decision-making, and anxious adolescents display atypical temporal coordination between prefrontal and parietal regions, which may underlie heightened avoidance under uncertainty. In this population, delayed maturation of prefrontal–striatal connectivity has been associated with reduced top–down regulation of the amygdala, resulting in the dominance of emotion-driven over goal-directed responses (41, 42). Moreover, excessive β- and θ-band synchronization within visual–emotional integration regions, such as the occipital–temporal cortex, may reflect compensatory recruitment of neural resources during the processing of potential threat and reward signals (24, 43, 44). Parietal regions contribute to uncertain decision-making by integrating attentional resources and outcome-related information. Parietal theta activity has been implicated in conflict monitoring and behavioral adjustment under conditions of ambiguity or potential loss. In addition, parietal–temporal interactions may facilitate the integration of attentional and emotional information during negative outcome evaluation (45). Given that heightened threat sensitivity and sustained monitoring of adverse outcomes are characteristic of anxiety, increased theta-band synchronization between parietal and temporal regions may represent enhanced neural coordination during loss-related uncertainty processing. Based on these observations, we hypothesized that, compared with non-anxious adolescents, anxious adolescents would exhibit enhanced β-band connectivity between occipital and temporal regions during gain-related uncertain decision-making, rwhich may reflect altered processing of uncertain reward-related information. Furthermore, we predicted that anxious adolescents would show increased θ-band connectivity between parietal and temporal regions during loss-related uncertain decision-making, reflecting enhanced processing of negative outcomes under uncertainty (46).

This study employs the Balloon Analogue Risk Task (BART) to investigate how anxiety influences adolescent decision-making under uncertainty (47, 48). The task manipulates gain and loss conditions to capture the differential impact of potential rewards and punishments, thereby simulating real-world uncertain decision scenarios (49). Behavioral measures, including pump attempts and task scores, are used to quantify both exploratory and avoidance tendencies in these contexts. Concurrent EEG time–frequency analyses focus on β (13–30 Hz) and θ (4–7 Hz) oscillations, which are implicated in cognitive control, reward processing, emotion regulation, and visual–emotional integration (31). In addition, EEG functional connectivity analyses quantify phase synchronization between regions supporting reward, cognitive control, and affective processing, capturing large-scale network interactions (50, 51). Prior research indicates that anxious adolescents exhibit delayed maturation of prefrontal–striatal circuits, resulting in reduced top–down regulation of the amygdala, while occipital–temporal regions show excessive β- and θ-band synchronization, potentially reflecting compensatory recruitment during threat and reward processing (41, 52). By integrating behavioral, oscillatory, and connectivity measures across gain and loss contexts, this study aims to elucidate how anxiety shapes adolescent decision-making under uncertainty. Specifically, we seek to link distinct patterns of β and θ activity and network connectivity to behavioral avoidance, thereby providing neurophysiological insights that could inform future intervention strategies.

## 2 Materials and methods

### 2.1 Participants

The sample size was estimated *a priori* using G*Power (53). For a repeated-measures design examining a within–between interaction (two groups), assuming an effect size of f = 0.28, α = .05, and power (1 − β) = .80, a minimum of 20 participants per group was required. We initially recruited 44 right-handed adolescents (22 with anxiety disorders and 22 healthy controls) from a single regional psychiatric clinic and local schools within the same geographic area. Four participants were excluded due to technical issues or incomplete task data, resulting in a final sample of 40 adolescents (20 per group).

The two groups were matched on key demographic variables. Independent-samples t-tests and a chi-square test indicated no significant differences in age (Anxiety: M = 16.2, SD = 1.5; Control: M = 15.9, SD = 1.4; t(38) = 0.68, p = .499), intelligence (Anxiety: M = 105.3, SD = 6.8; Control: M = 106.7, SD = 7.2; t(38) = −0.65, p = .521), or sex distribution (Anxiety: 12 females/8 males; Control: 11 females/9 males; χ²(1) = 0.10, p = .752).

Adolescents in the anxiety group were recruited from the outpatient clinic of a regional psychiatric hospital. Clinical diagnoses were established according to the International Classification of Diseases, 10th Revision (ICD-10) criteria through structured clinical interviews conducted by licensed child and adolescent psychiatrists. Diagnoses were confirmed based on clinical interviews and medical record review. The anxiety group consisted of adolescents with clinically significant anxiety disorders, including clinically diagnosed anxiety disorders. The present study focused on anxiety-related alterations in uncertainty processing rather than differences among specific anxiety subtypes.

Clinical characteristics and eligibility criteria for the anxiety group: (1) ICD-10 diagnosis of a moderate-to-severe anxiety disorder; (2) age between 14 and 17 years; this age range was selected to capture mid-to-late adolescence, a developmental stage characterized by ongoing maturation of cognitive control and emotion regulation systems. Restricting the age range was intended to reduce developmental heterogeneity and minimize potential confounding effects associated with age-related differences in neural maturation and decision-making processes; (3) Clinically significant anxiety symptoms were assessed using the Generalized Anxiety Disorder-7 (GAD-7; 54). The GAD-7 was used as a dimensional measure of anxiety symptom severity rather than a diagnostic instrument, as diagnoses were established through clinical interviews based on ICD-10 criteria. A score ≥10 was considered indicative of moderate-to-severe anxiety symptoms. Although the GAD-7 primarily captures generalized anxiety symptoms, it provides a standardized assessment of worry-related symptom severity relevant to uncertainty processing; (4) right-handedness and normal color vision; (5) first-episode and treatment-naïve status; and (6) written informed consent from both the participant and a legal guardian. Exclusion criteria included: (1) primary psychiatric comorbidity; (2) history of neurological disorders; (3) contraindications to study procedures; and (4) IQ< 80. Intellectual functioning was assessed using the Wechsler Intelligence Scale for Children (WISC-V), which was administered by trained professionals prior to EEG recording.

Healthy controls were recruited from local middle and high schools within the same region through informational sessions, school announcements, and study flyer distribution. Interested families contacted the research team for a telephone screening to assess initial eligibility. Controls were matched to the anxiety group by age (± 1 year) and sex. Inclusion criteria were: (1) no current or lifetime psychiatric diagnosis as determined by structured clinical interview; (2) subclinical anxiety levels (GAD-7< 5); (3) age 14–17 years; (4) right-handedness and normal color vision; (5) no family history of major psychiatric disorders; and (6) written informed consent. The same exclusion criteria applied to the anxiety group, including neurological disorders, contraindications to study procedures, and IQ< 80, were also applied to healthy controls to ensure comparability between groups.

The study was approved by the institutional ethics committee (approval No. IRB2025081, September 29, 2025) and conducted in accordance with the Declaration of Helsinki. All participants and their guardians provided written informed consent prior to participation and received modest financial compensation.

### 2.2 Tasks and procedures

#### 2.2.1 Balloon Analogue Risk Task (BART) procedure

The Balloon Analogue Risk Task (BART) was used to assess decision-making under uncertainty (47, 48; **Figure 1**). Participants received instructions and a practice session.

**Figure 1:**
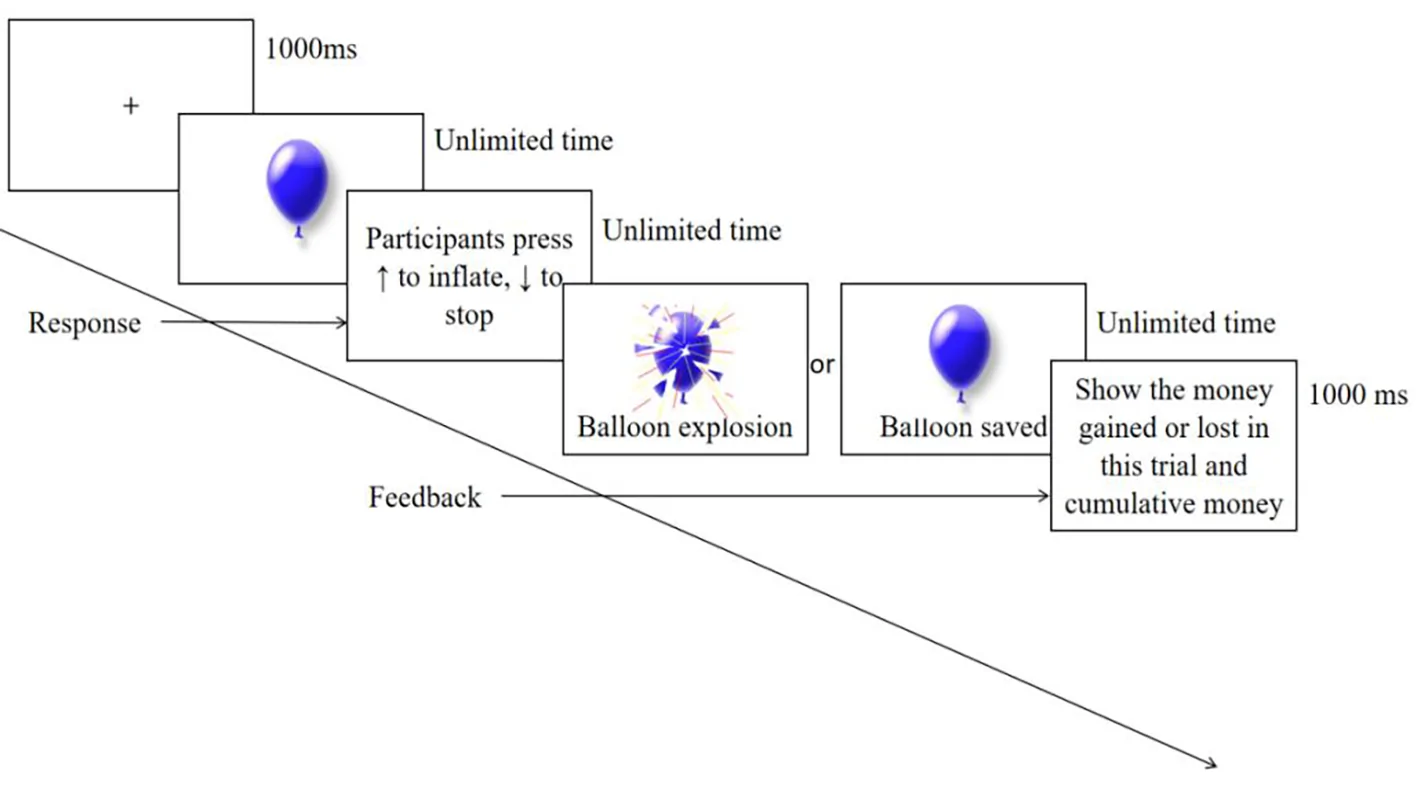
Trial sequence of the balloon analogue risk task (BART).

Each trial began with a 1000-ms fixation cross, followed by the presentation of a virtual balloon. Participants pressed an up-arrow key to inflate the balloon; each press increased its size slightly. During the balloon inflation phase, participants were allowed unlimited time to decide whether to continue pumping or stop to secure the accumulated outcome. Once participants chose to stop pumping or the balloon exploded, outcome feedback (gain/loss for the trial and cumulative total) was displayed for 1000 ms.

Gain Condition: Each pump added ¥0.05 to the trial’s potential gain. Each balloon had a hidden explosion point (randomly set between 1–64 pumps). The maximum gain per balloon was ¥3.20. Participants could cash out at any time to secure the accumulated gain; if the balloon exploded, the trial’s earnings were lost. Loss Condition: Each balloon started with a potential loss of ¥3.20. Each pump reduced the loss by ¥0.05. The explosion risk was identical to the Gain condition. Participants could stop to finalize the reduced loss; an explosion incurred the full ¥3.20 loss.

The underlying explosion probability was identical across conditions; only the framing (accumulating gains vs. avoiding losses) and feedback wording differed. After two practice trials, each participant completed 30 Gain-frame and 30 Loss-frame trials. Block order was counterbalanced.

#### 2.2.2 Behavioral measures

Behavioral measures recorded during the BART included the number of pump attempts, cash-out decisions, explosion frequency, and BART score. The BART score was selected as the primary behavioral outcome because it provides an integrated measure of risk-taking behavior by reflecting participants’ willingness to pursue potential rewards while considering the risk of balloon explosion. Although additional behavioral variables were recorded for descriptive purposes, only BART scores were entered into the primary statistical analyses to quantify individual differences in decision-making under uncertainty.

### 2.3 EEG recording

EEG data were recorded using a 32-channel wireless system (StEEG Vega, Artise Biomedical). Saline-soaked sponge sensors were mounted on a fabric cap according to the 10–20 system. The online reference was REF, with GND as ground; data were later re-referenced to the average of the mastoids. Impedance was kept below 25 kΩ, consistent with manufacturer specifications for this electrode type and supported by validation studies. Data were sampled at 500 Hz and transmitted wirelessly. Behavioral responses were recorded via E-Prime 3.0.

### 2.4 EEG data preprocessing

Data were segmented into epochs from −1000 ms to +1000 ms relative to the onset of outcome feedback. Feedback events were defined as successful cash-out (gain outcome) or balloon explosion (loss outcome). Thus, EEG analyses were feedback-locked, focusing on neural responses related to outcome evaluation and uncertainty processing. Independent component analysis (ICA) was performed using the extended Infomax algorithm implemented in EEGLAB to identify and remove artifacts. Components reflecting ocular and muscle artifacts were manually identified based on scalp topography, time-course characteristics, and power spectral density. Ocular components were characterized by frontal scalp distributions and blink- or saccade-related activity, whereas muscle components showed focal scalp distributions with increased high-frequency activity (>30 Hz). On average, 3.8 (SD = 1.4) components were removed per participant.

Epochs containing residual artifacts exceeding ±100 μV at any electrode were automatically rejected. After artifact rejection, an average of 90.3% of trials per participant were retained (range: 64.5%–98.4%). A participant-level analysis revealed that a subset of participants retained fewer than 75% of trials, exceeding the 25% rejection cutoff recommended by Luck (22). However, retention rates were comparable between groups, and no systematic group differences in data quality were observed. To ensure the robustness of our findings, we reanalyzed the primary EEG measures after excluding these participants. This supplementary analysis yielded a pattern of results consistent with our main findings, confirming that our conclusions were not substantively influenced by the inclusion of participants with higher rejection rates. All participants retained more than 60% of trials; however, we adopted the more conservative 75% threshold in our supplementary analysis to directly address the recommended standard (22).

### 2.5 Time–frequency analysis

#### 2.5.1 Frequency band definition and rationale

The analysis focused on three canonical frequency bands: theta (θ, 4–8 Hz), alpha (α, 8–13 Hz), and beta (β, 13–30 Hz). These standard frequency ranges were selected based on extensive prior literature demonstrating their robust and consistent association with neurocognitive processes central to decision-making across development, including adolescence. Specifically, theta activity has been linked to conflict monitoring and cognitive control, alpha to attentional and inhibitory processes, and beta to cognitive control, sensorimotor integration, and reward-related processing. We acknowledge that individual spectral properties, such as the individual alpha peak frequency (IAF), may vary across individuals and undergo developmental shifts. Although individualized frequency band approaches can enhance sensitivity, the use of canonical frequency bands facilitates comparability and reproducibility across studies. Given that the primary aim of the present study was to examine group-level differences between anxious and non-anxious adolescents and to situate our findings within the broader decision-making literature—most of which employs standard frequency definitions—the use of canonical bands was considered methodologically appropriate.

#### 2.5.2 Decomposition and statistical analysis

EEG analyses were feedback-locked to the onset of outcome feedback to characterize neural responses related to outcome evaluation and uncertainty processing. Event-related spectral perturbation (ERSP) was calculated from artifact-free epochs using a complex Morlet wavelet transform implemented in EEGLAB’s newtimef function. Wavelet cycles increased linearly from 3 cycles at 1 Hz to 10 cycles at 45 Hz to balance temporal and spectral resolution (23). EEG epochs were extracted from −1000 to +1000 ms relative to feedback onset, including balloon explosions (loss outcomes) and successful cash-outs (gain outcomes). Time–frequency power was normalized using a decibel transformation relative to a pre-feedback baseline interval (−800 to −100 ms), calculated as 10 × log10(power/baseline).

The processed ERSP data were analyzed using a repeated-measures analysis of variance (rmANOVA), with Group (Anxious, Healthy Control) as a between-subjects factor and Framing (Gain, Loss) as a within-subjects factor. To correct for multiple comparisons across the time–frequency matrix, a cluster-based permutation correction was applied (cluster-level p<.05). Significant clusters were identified based on spectral-temporal contiguity criteria, including frequency continuity and temporal persistence. Specifically, a cluster was considered reliable if it met the following criteria: (1) Frequency continuity: significant effects within a frequency band extended across at least two consecutive time points with at least one overlapping significant time point; and (2) Temporal persistence: significant activity persisted for at least ten consecutive time points across two or more adjacent frequency bins.

Correlation analyses were conducted to examine the associations between task-related EEG connectivity and behavioral performance. Specifically, two hypothesis-driven Pearson correlation analyses were performed within the anxiety group: β-band occipito-temporal connectivity during gain feedback and θ-band parietal–temporal connectivity during loss feedback were correlated with BART scores. Because these analyses were limited to two predefined correlations based on *a priori* hypotheses, no additional correction for multiple comparisons was applied. All correlations were two-tailed, with statistical significance set at *p*<.05.

### 2.6 Functional connectivity analysis

#### 2.6.1 Electrode pair selection

The selection of electrode pairs for connectivity analysis was guided primarily by theoretical considerations regarding the neural processes involved in uncertain decision-making. Specifically, we focused on task-related occipito-temporal and parietal–temporal functional interactions because occipital and temporal regions contribute to the integration of perceptual and contextual information, whereas parietal–temporal interactions are involved in attentional allocation, outcome evaluation, and affective processing during decision-making (35, 55). These processes are particularly relevant to uncertain choices involving potential gains and losses (56, 57). Importantly, the selected electrode pairs do not represent predefined resting-state networks but rather reflect task-related EEG functional interactions measured during the BART. Based on this theoretical framework, the occipito-temporal connection (O2–TP7) in the β band and the parietal–temporal connection (P4–P7) in the θ band were selected for hypothesis-driven analyses. Because source reconstruction was not performed, these electrode pairs are interpreted as scalp-recorded electrophysiological interactions rather than direct indicators of activity originating from specific cortical regions.

#### 2.6.2 Connectivity metric and volume conduction mitigation

Phase-based functional connectivity was assessed using the phase-locking value (PLV) to quantify task-related neural synchronization. Raw PLV values were calculated to characterize the consistency of phase synchronization between two signals across trials, with values ranging from 0 (random phase relationships) to 1 (perfect phase locking). PLV is widely used in EEG connectivity research due to its sensitivity to task-related changes in inter-regional communication (23, 58, 59). The instantaneous phase ϕ(t,f) was extracted for each trial using the same Morlet wavelet transform applied in the time-frequency analysis (Section 2.5.1) to ensure methodological consistency.

To mitigate the spurious connectivity estimates caused by volume conduction—a well-known confound in scalp EEG where a single source is picked up by multiple electrodes (60, 61)—a current source density (CSD) transform was applied to the epoched data prior to connectivity estimation. The CSD transform acts as a spatial filter that emphasizes local, radial current sources and suppresses far-field, volume-conducted activity, thereby enhancing the spatial specificity of phase estimates (62, 63). The effectiveness of this procedure was confirmed by observing a marked reduction in zero-lag correlation between neighboring electrodes post-CSD. For each electrode pair i and j, the raw PLV was computed as follows:


PLV_ ij(t,f)=|(1/N)∑_{n=1}^{N} exp[i˙Δϕ_ij(t,f,n) ]|

where N represents the number of trials, and Δϕij(t,f,n) denotes the instantaneous phase difference between electrodes i and j at time t and frequency f for trial n. To account for intrinsic baseline differences in phase synchronization, baseline correction was performed by subtracting the mean PLV during the pre-feedback baseline interval (−750 to −250 ms) from task-related PLV values. Baseline-corrected PLV values were subsequently extracted within the predefined frequency and temporal windows and used for all statistical analyses. Time windows for connectivity analyses were defined based on significant task-related oscillatory clusters identified in the time–frequency analysis. Specifically, β-band connectivity was analyzed within the 100–400 ms interval after feedback onset, whereas θ-band connectivity was examined within the 600–900 ms interval after feedback onset. Baseline-corrected PLV values were extracted within these predefined frequency and temporal windows for subsequent statistical analyses.

## 3 Result

### 3.1 Behavioral result

The anxious and healthy control groups were successfully matched on key demographic and cognitive baseline characteristics. Independent-samples t-tests confirmed that there were no significant differences between groups in age (*t*(38) = 0.68, *p* = .499) or intelligence quotient (*t*(38) = -0.65, *p* = .521). Furthermore, a chi-square test indicated that the groups did not differ in sex distribution (*χ²*(1) = 0.10, *p* = .752).

A mixed-design analysis of variance (RMANOVA) was conducted to examine the effects of participant type (anxious vs. non-anxious) and framing (gain vs. loss contexts) on decision-making under uncertainty. The dependent variable was the mean BART score calculated separately for each condition, with participant type entered as a between-subjects factor and framing as a within-subjects factor. The main effect of framing was not significant, *F*(1, 38) = 0.015, *p* = 0.904, *ηp²* = 0.000. The main effect of participant type was significant, *F*(1, 38) = 26.018, *p*< 0.001, *ηp²* = 0.255, with anxious adolescents exhibiting lower BART scores than non-anxious adolescents (M ± SD = 31.95 ± 3.20 vs. 55.02 ± 3.20).The interaction between participant type and framing was also significant, *F*(1, 38) = 4.683, *p* = 0.034, *ηp²* = 0.058.*Post hoc* independent-samples t-tests were conducted to decompose the interaction. Results revealed that during gain-related feedback processing under uncertainty, anxious adolescents (M = 37.12, SD = 18.22) exhibited significantly lower BART scores than non-anxious adolescents (M = 50.40, SD = 15.66), t(38) = 2.11, p = 0.041, Cohen’s d = 0.78, 95% CI [0.04, 1.52]. Similarly, during loss-related uncertain decision-making, anxious adolescents (M = 26.78, SD = 8.10) showed significantly lower scores compared with non-anxious adolescents (M = 59.64, SD = 31.53), t(38) = 4.02, p< 0.001, Cohen’s d = 1.42, 95% CI [0.73, 2.11];see **Table 1**. These patterns are illustrated in **Figure 2**, which shows the distribution of BART scores across groups and frames, with anxious adolescents consistently displaying reduced more conservative decision-making strategy under uncertainty, as indicated by lower BART scores, particularly in the loss frame.

**Table 1:** Descriptive statistics of BART scores by frame and participant type.

Frame	Participant type	M	SD	Participant type
Gain	Anxious	37.12	18.22	Anxious
Gain	Non-anxious	50.40	15.66	Non-anxious
Loss	Anxious	26.78	8.10	Anxious
Loss	Non-anxious	59.64	31.53	Non-anxious

**Figure 2:**
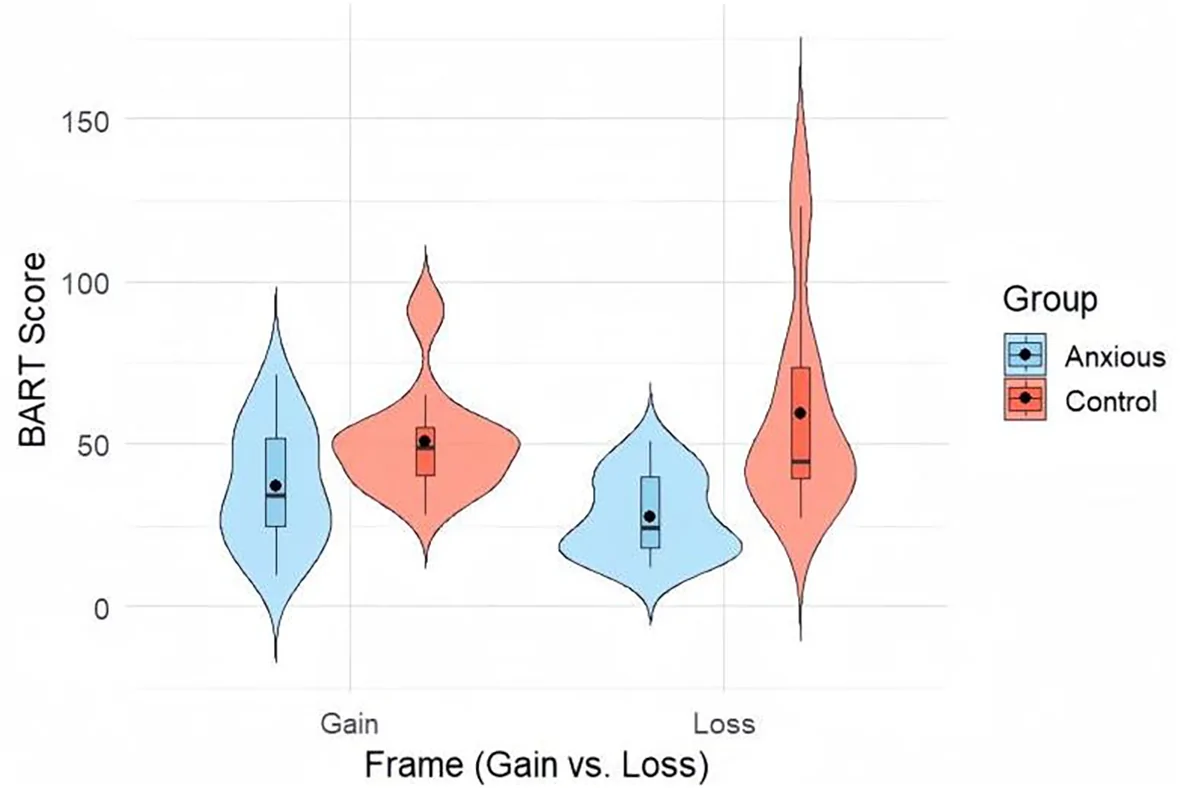
Violin plots of BART scores by participant type (anxious vs. non-anxious) and framing condition (gain vs. loss).

### 3.2 Time-frequency results

Analysis revealed that the main effect of framing in the β frequency range was not significant [*F*(1,38) = 0.008, *p* = 0.929]. A significant main effect of participant type was observed [*F*(1,38) = 6.189, *p* = 0.017, *ηp²* = 0.140], with anxious adolescents showing higher overall β power than non-anxious adolescents when the two framing conditions were considered together (M ± SD = 0.199 ± 0.083 vs. −0.094 ± 0.083). However, this main effect should be interpreted in conjunction with the significant Group × Frame interaction [*F*(1,38) = 4.878, *p* = 0.033, *ηp²* = 0.114], which indicates that the magnitude and direction of the group difference varied across framing conditions. Specifically, during gain-related feedback processing, anxious adolescents showed lower β power than non-anxious adolescents (M = 0.228, SD = 0.163 vs. M = 0.323, SD = 0.030), *t*(38) = 2.38, *p* = 0.022, Cohen’s d = 0.76. Thus, the significant group main effect reflects the overall difference averaged across the two framing conditions and does not imply uniformly higher β power in anxious adolescents in each condition. Rather, the significant interaction indicates that β-band activity was modulated by decision framing, with the clearest group difference occurring during gain-related processing (see **Table 2**; **Figures 3**, **4**).

**Table 2:** Descriptive statistics of Time-frequency by frame and participant type.

Frame	Participant type	M	SD
Gain	Anxious	0.228	0.163
Gain	Non-anxious	0.323	0.030
Loss	Anxious	0.041	0.112
Loss	Non-anxious	0.747	0.152

**Figure 3:**
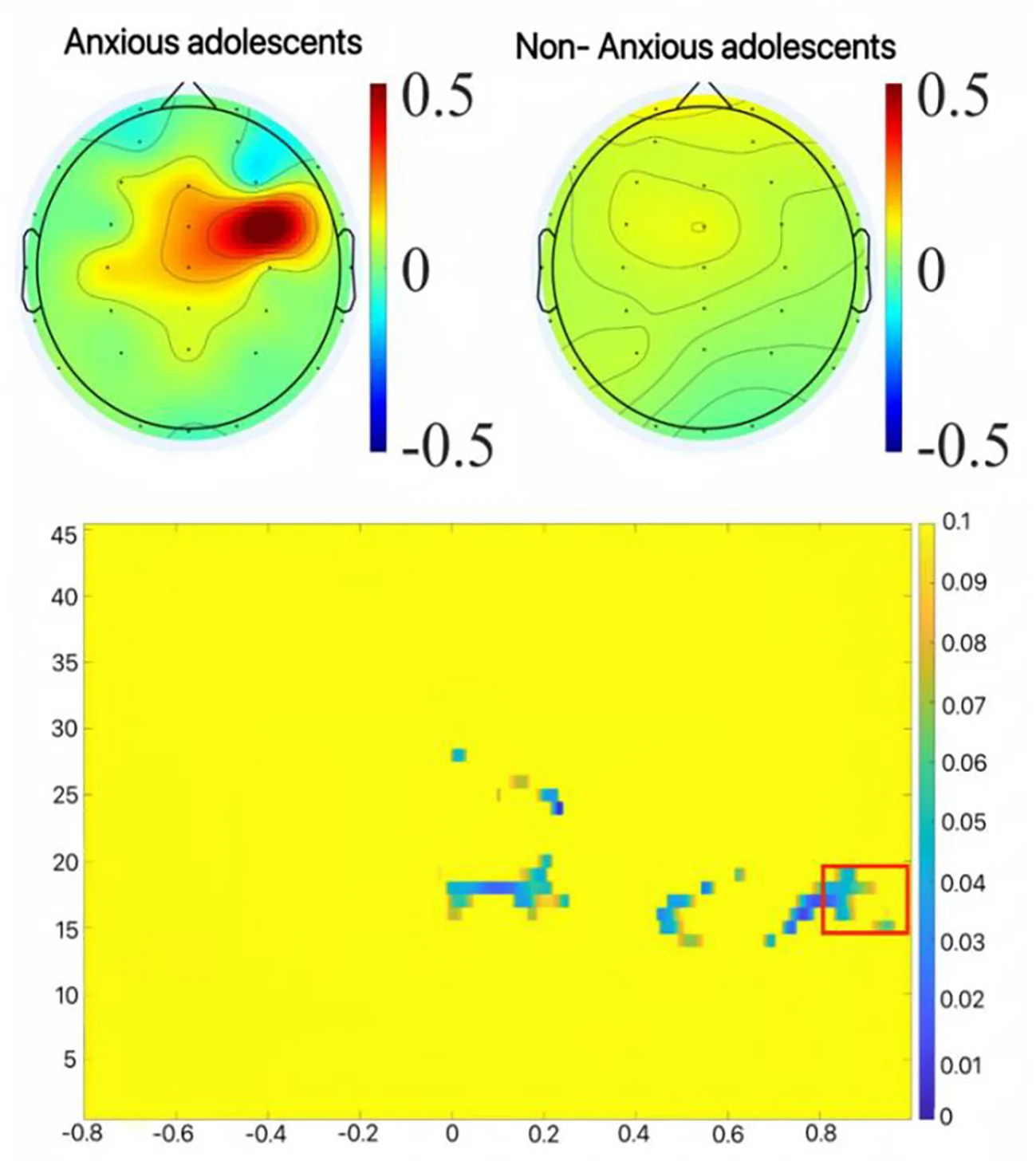
Time–frequency analysis results of β-band activity in anxious and non-anxious adolescents. The spectrogram displays the distribution of significant event-related spectral perturbation (ERSP) values. Rectangles indicate regions of interest showing significant time–frequency patterns. A significant main effect of group was observed in β-band event-related synchronization within the 0.8–1 s time window.

**Figure 4:**
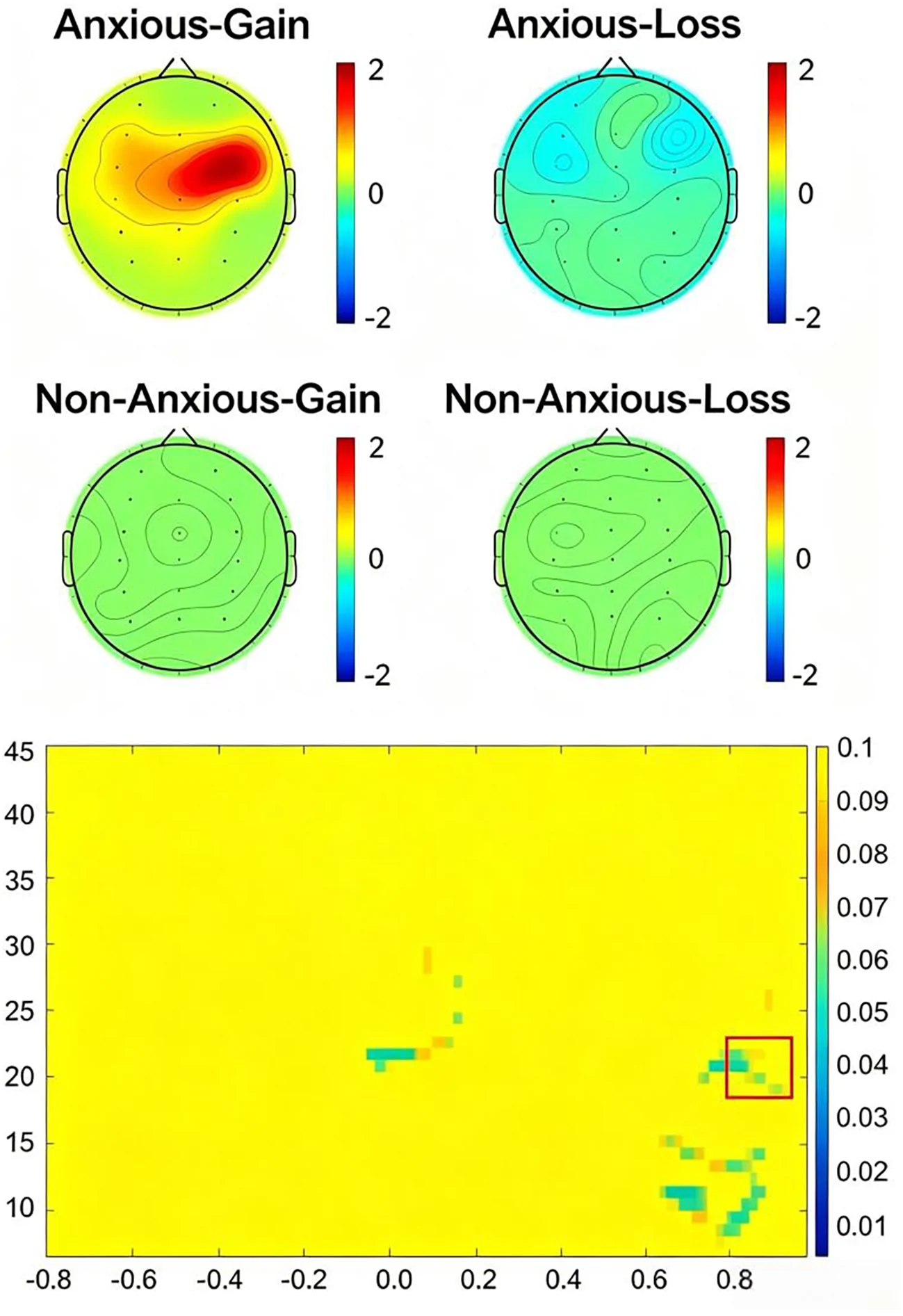
Time–frequency analysis results of β-band activity across the whole brain. The topographic maps illustrate significant event-related spectral perturbation (ERSP) values in both groups (anxious vs. non-anxious adolescents) and across framing conditions (gain vs. loss). Regions of interest (ROIs) with significant time–frequency patterns are highlighted with rectangles. Specifically, a significant interaction effect was observed in β-band ERSP within the 0.7–1 s time window.

### 3.3 Functional connection analysis result

Connectivity analysis revealed a significant interaction effect in the beta band (100–400 ms) between occipital and temporal electrodes (O2–TP7). The analysis indicated that the main effects of framing [*F*(1,38) = 0.425, *P* = 0.518] and participant type [*F*(1,38) = 1.981, *P* = 0.167] were not significant. However, a significant interaction between participant type and framing effect was found [*F*(1,38) = 6.150, *P* = 0.018]. Follow-up comparisons indicated higher β-band PLV during gain-related feedback processing in anxious adolescents compared with non-anxious adolescents (M ± SD = 0.02 ± 0.01 vs. −0.019 ± 0.010, *p* = 0.009; see **Table 3**; **Figure 5**).

**Table 3:** Descriptive statistics of PLVs in β and θ bands by frame and participant type.

Frame	Participant type	β-band PLV		θ-band PLV	
		M	SD	M	SD
Gain	Anxious	0.021	0.042	-0.024	0.052
Gain	Non-anxious	-0.019	0.049	-0.006	0.051
Loss	Anxious	-0.005	0.044	0.027	0.066
Loss	Non-anxious	-0.004	0.065	-0.035	0.055

**Figure 5:**
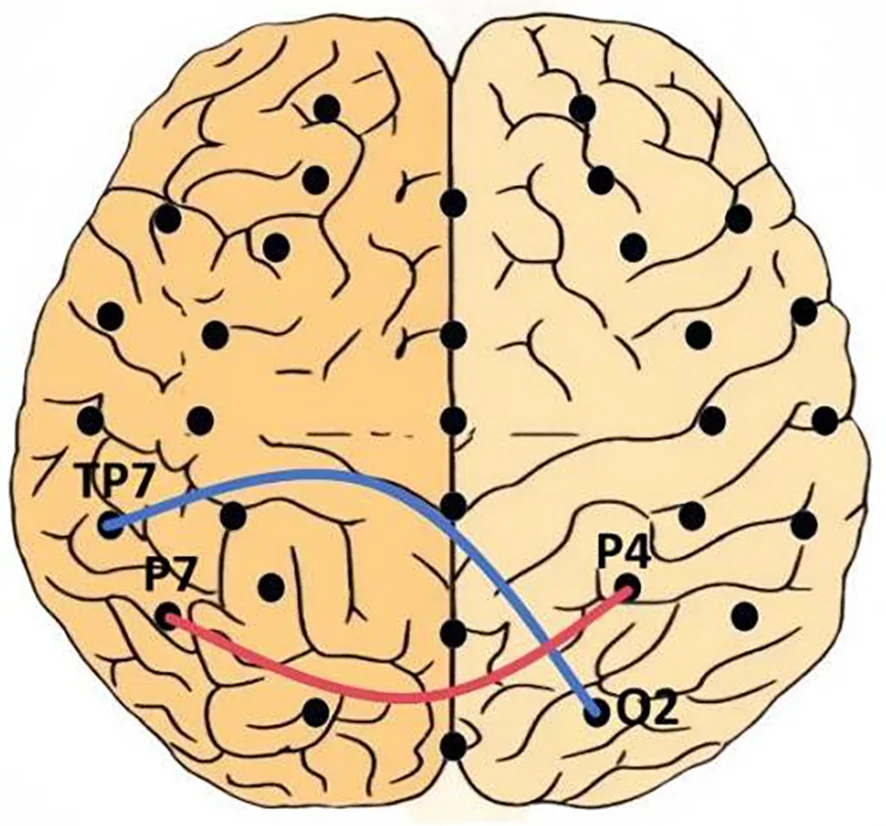
Topographic representations of significant functional connectivity differences based on phase-locking values (PLVs). In the β band (100–400 ms), significant connectivity was observed between the occipital (O2) and temporal (TP7) regions, showing a significant interaction between participant type and framing effect. In the θ band (600–900 ms), significant connectivity was observed between the parietal (P4) and temporal (P7) regions, also showing a significant interaction effect. Nodes represent recording sites (electrodes).

Exploratory connectivity analyses identified a theta-band interaction effect in the theta band (600–900 ms) between the parietal and temporal regions (P4–P7). Repeated-measures ANOVA with average PLV as the dependent variable showed that the main effects of framing [*F*(1,38) = 1.083, *P* = 0.305] and participant type [*F*(1,38) = 2.350, *P* = 0.134] were not significant. However, a significant interaction between framing effect and participant type was found [*F*(1,38) = 14.163, *P* = 0.001, *ηp²* = 0.272]. Further simple effects analysis revealed that, during loss-related uncertain decision-making, the brain-phase-locking value (PLV) of anxious adolescents (M ± SD = 0.03 ± 0.01) was higher than that of non-anxious adolescents (M ± SD = 0.03 ± 0.01 vs. -0.04 ± 0.01, *p* = 0.003; see **Table 3**; **Figure 5**). Summaries of the significant EEG findings are provided in **Tables 4** and **5**. **Table 4** summarizes the time–frequency results, whereas **Table 5** summarizes the functional connectivity findings.

**Table 4:** Summary of significant time–frequency EEG findings.

EEG measure	Participant type	Electrode(s)	Time window	Contrast	Direction of effect	p-value	Effect size
Time–frequency analysis	β (13–30 Hz)	Significant scalp cluster	700–1000 ms	Anxiety vs. Control	Higher overall β power in anxious adolescents	0.017	*ηp²* = 0.140
Time–frequency analysis	β (13–30 Hz)	Significant scalp cluster	700–1000 ms	Group × Frame	Lower β power in anxious adolescents during gain-related processing	0.033	*ηp²* = 0.114

**Table 5:** Summary of significant functional connectivity findings.

EEG measure	Frequency band	Electrode pair	Time window	Contrast	Direction of effect	p-value	Effect size
Functional connectivity	β (13–30 Hz)	Significant scalp cluster	100–400 ms	Group × Frame	Higher β-band PLV during gain in anxious adolescents	0.018	*ηp²* = 0.139
Functional connectivity	θ (4–7 Hz)	Significant scalp cluster	600–900 ms	Group × Frame	Higher θ-band PLV during loss in anxious adolescents	0.001	*ηp²* = 0.272

### 3.4 Relevant analysis results

To further examine the behavioral relevance of oscillatory connectivity, Spearman rank correlation analyses were conducted between BART performance and PLVs under gain and loss frames within the anxious group. Two hypothesis-driven correlations were tested, including the association between θ-band PLV during loss-related decision-making and BART scores, and the association between β-band PLV during gain-related decision-making and BART scores. Given the limited number of *a priori* specified correlations, p-values were interpreted without additional multiple-comparison correction.

Under the loss frame, BART scores were negatively associated with θ-band PLV between the parietal and temporal regions (rs = −0.482, *p* = 0.031, 95% CI [−0.73, −0.05]), indicating that higher θ-band synchronization was associated with more conservative decision-making behavior. Similarly, under the gain frame, BART scores were negatively associated with β-band PLV between the occipital and temporal regions (*r* = −0.506, *p* = 0.023), suggesting that increased β-band connectivity was associated with reduced risk-taking tendencies (see **Figure 6**). These findings indicate that task-related oscillatory coordination is associated with individual differences in uncertainty-related decision behavior, supporting a potential relationship between β-band regulatory processes, θ-band monitoring mechanisms, and anxiety-related decision biases.

**Figure 6:**
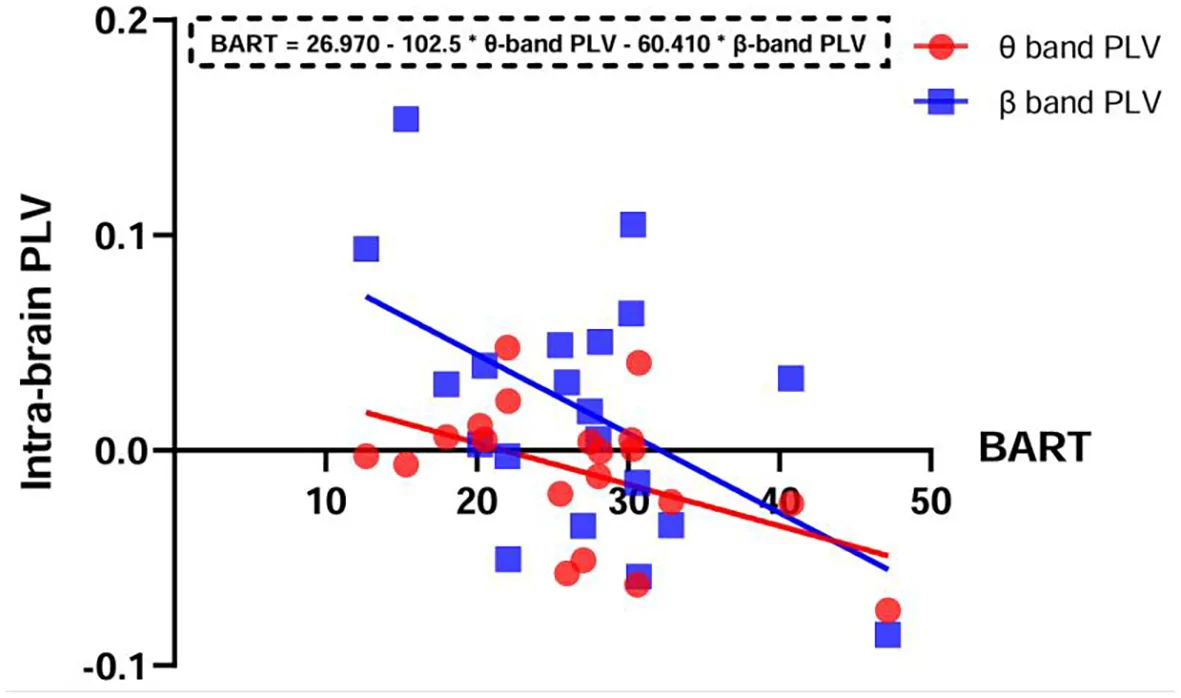
Scatter plot of correlation.

### 3.5 Regression analysis results

To further examine whether oscillatory connectivity was associated with behavioral performance during uncertain decision-making, a linear mixed-effects regression analysis was conducted with BART scores as the dependent variable and β- and θ-band PLVs as predictors. The results showed that θ-band PLV between the parietal and temporal electrodes was negatively associated with BART scores (*t* = −2.187, *p* = 0.043), and β-band PLV between the occipital and temporal electrodes was also negatively associated with BART scores (*t* = −2.341, *p* = 0.032). The overall regression model explained 41.9% of the variance in BART performance (*R²* = 0.419, *p* = 0.010). These findings suggest that task-related oscillatory connectivity is associated with individual differences in uncertainty-related decision-making behavior.

## 4 Discussion

Decision-making is a critical aspect of adolescents’ cognitive and socio-emotional development, shaped by the interaction of neural mechanisms, psychological states, and contextual influences (64). Compared to non-anxious adolescents, those with anxiety exhibit stronger avoidance tendencies under uncertainty, biased information processing, and reduced prefrontal cortical regulatory capacity, impairing their ability to weigh potential gains and losses during decision-making (65). In the present study, behavioral measures were integrated with EEG time–frequency and functional connectivity analyses using the Balloon Analogue Risk Task (BART) to compare anxious and non-anxious adolescents across gain and loss contexts. Behavioral results indicated that anxious adolescents demonstrated more conservative decision-making under uncertainty, as reflected by significantly lower BART scores and fewer pump attempts. Time–frequency analyses revealed that β-band activity differed between anxious and non-anxious adolescents depending on decision framing. Specifically, anxious adolescents showed reduced β power during gain-related uncertain decision-making, whereas no significant group difference was observed during loss framing. Functional connectivity analyses further showed that, in the β band, anxious adolescents exhibited stronger phase synchronization in the gain context, suggesting altered neural coordination during uncertain reward evaluation. In the θ band, they displayed increased parietal–temporal connectivity during loss-related feedback processing, which may reflect enhanced monitoring and evaluation of negative outcomes under uncertainty. Collectively, these findings suggest that anxiety is associated with a more cautious decision-making style under uncertainty, accompanied by alterations in β- and θ-band oscillatory activity and connectivity patterns. Crucially, these electrophysiological alterations should be interpreted within the developmental context of adolescence, a period characterized by ongoing maturation of large-scale cognitive control and affective systems (66, 67). Thus, the observed patterns likely reflect an interaction between anxiety pathology and the developmental state of the cognitive control system.

The behavioral results indicated that anxious adolescents demonstrated more conservative decision-making under uncertainty in both gain and loss contexts. They chose to “cash out” earlier and had significantly fewer pump responses compared to their non-anxious peers (68). This finding is consistent with prior research suggesting that the adolescent prefrontal cortex is not fully matured, particularly in regions responsible for executive functions and emotional regulation, leading to inefficiency in processing ambiguous information. Anxiety may amplify this developmental characteristic, making adolescents more prone to conservative choices in uncertain contexts (69). Anxious adolescents typically exhibit lower self-efficacy, a trait that may influence their evaluation and choice in uncertain situations. Individuals with higher self-efficacy are less likely to adopt avoidance strategies, whereas those with lower self-efficacy may experience heightened anxiety, resulting in more cautious choices (70, 71). Therefore, the conservative behavior observed may be attributed not only to neural developmental and emotion regulation deficiencies but also to the influence of lower self-efficacy.

β-band activity differed between anxious and non-anxious adolescents during uncertainty-based decision-making, with the direction of group differences depending on decision framing (40). Importantly, although a significant main effect of participant type indicated higher overall β power in anxious adolescents when the gain and loss conditions were considered together, this effect was further qualified by a significant Group × Frame interaction. This interaction indicates that the group difference in β power was not consistent across framing conditions. Specifically, anxious adolescents showed lower β power than non-anxious adolescents during gain-related feedback processing, whereas no significant group difference was observed during loss framing. Thus, the significant main effect should be understood as reflecting the average group difference across the two framing conditions, rather than as a uniform increase in β power in anxious adolescents across both contexts. This framing-dependent pattern suggests that anxiety-related alterations in β-band activity may be sensitive to the motivational context of uncertain decision-making. The observed modulation occurred during the 700–1000 ms period following feedback onset, which may reflect later-stage processing involved in the evaluation and regulation of uncertain outcomes. Given that β oscillations (13–30 Hz) have been associated with cognitive control, attentional regulation, and top-down maintenance of task-relevant representations (24, 25, 30), the reduced β power observed in anxious adolescents during gain-related processing may reflect altered neural engagement during the evaluation of uncertain rewards and regulation of subsequent responses (72). Importantly, this interpretation reflects a framing-dependent alteration in β-band activity rather than a generalized reduction in β power, because the group difference was not significant under the loss frame. This context-dependent modulation is consistent with previous evidence that motivational context can influence β-band dynamics during decision-making (73). Specifically, the altered β-band response observed in anxious adolescents during gain-related processing may reflect differences in the recruitment of cognitive control processes when evaluating uncertain rewards. Prior EEG studies have shown that β oscillations are sensitive to reward-related feedback and are prominent in prefrontal regions following positive outcomes (74), contributing to the maintenance of task-relevant representations beyond outcome valence (75). Developmentally, altered engagement of these mechanisms may align with evidence that anxious adolescents exhibit stronger processing biases, which may place greater demands on cognitive control systems (76). Overall, context-dependent alterations in β dynamics may represent a potential neural correlate of anxiety-related decision-making biases under uncertainty (77), providing further evidence for the potential involvement of top-down regulatory processes and highlighting β oscillations as a potential neurophysiological correlate of anxiety-related alterations in uncertain decision-making (78).

Examination of functional connectivity revealed distinct scalp-recorded electrophysiological patterns. In the β band (100–400 ms after feedback onset), a significant group-by-framing interaction was observed in occipito-temporal (O2–TP7) connectivity, with anxious adolescents showing stronger connectivity during gain-related feedback processing. This increased occipito-temporal scalp-level β synchronization may be associated with altered coordination of perceptual and affective-contextual information processing during ambiguous reward-related feedback (79, 80), potentially reflecting differences in sensitivity to uncertain reward outcomes. This interpretation is consistent with evidence that β oscillations are involved in uncertainty processing and the coordination between evaluative and regulatory processes (81, 82). Our findings extend previous work by suggesting that altered β-band synchronization in anxious adolescents may also emerge during reward-related uncertainty, providing a potential electrophysiological basis for uncertainty-avoidant decision-making strategies (83). In the θ band (600–900 ms after feedback onset), anxious adolescents displayed stronger parieto-temporal (P4–P7) scalp-level connectivity during loss-related feedback processing. This later temporal window is typically associated with outcome evaluation and adaptive adjustment. Increased θ-band synchronization between these posterior electrode sites may reflect enhanced coordination of attentional and affective-contextual processing during the evaluation of negative outcomes under uncertainty. This pattern may also be associated with increased cognitive demands during the processing and evaluation of negative outcomes, potentially involving conflict monitoring and behavioral adjustment. The dual pattern—enhanced β-band connectivity (PLV) during reward-related feedback and increased θ synchronization during loss-related feedback (84, 85)—suggests a generalized tendency toward uncertainty avoidance, potentially supported by heightened monitoring of uncertain and negative outcomes (86).

A key finding of this study is that oscillatory coordination between the scalp-recorded electrode pairs showing β- and θ-band modulation was not only group-differentiating but also behaviorally relevant. Correlation analyses demonstrated that, within the anxious group, stronger θ-band connectivity during loss contexts and enhanced β-band connectivity during gain contexts were significantly associated with lower BART scores, indicating more conservative decision-making. These negative associations provide more direct evidence that large-scale oscillatory synchronization co-varies with avoidance-related behavioral tendencies under uncertainty. Consistent with prior evidence linking β oscillations to vigilance and top-down control processes (25, 87–89) and θ connectivity to performance monitoring and threat processing (90, 91), increased β coupling during gains may be associated with greater engagement of regulatory processes, whereas enhanced θ connectivity during losses may be associated with sustained monitoring of potentially negative outcomes and affective-contextual processing (92).

Importantly, these neural–behavioral relationships should be interpreted within the developmental context of adolescence. Distributed cortical circuits supporting cognitive control, affective regulation, and long-range communication undergo substantial structural refinement during adolescence, including synaptic pruning and progressive myelination, which shape functional connectivity and oscillatory coordination (63, 66, 93). Heightened β and θ synchronization in anxious adolescents may therefore reflect an interaction between anxiety-related vulnerability and ongoing maturation of cognitive control and affective systems (76), rather than a fixed pathological deficit. Given the cross-sectional and correlational design, these findings identify neural signatures associated with avoidance behavior but do not establish causal pathways. Longitudinal and multimodal investigations will be necessary to clarify whether these oscillatory patterns represent antecedents, consequences, or dynamic correlates of adolescent anxiety.

Given the cross-sectional design, we cannot determine whether altered oscillatory activity represents a neural vulnerability marker or a consequence of prolonged anxiety. Longitudinal studies tracking oscillatory dynamics across adolescence would clarify developmental trajectories and causal relationships. Another limitation is the absence of real-time emotional state assessment during the task. Fluctuations in state anxiety or emotional arousal may dynamically modulate oscillatory activity and decision behavior. Future studies incorporating momentary affect ratings or physiological indices would help disentangle trait and state influences.

## 5 Limitations and future directions

The present study provides valuable insights into the neural mechanisms underlying decision-making in anxious adolescents; however, several limitations should be considered. First, the cross-sectional design limits causal inference and prevents clear differentiation between anxiety-related neural alterations and normative neurodevelopmental changes, particularly given that distributed cortical networks involved in cognitive control and affective regulation continue to mature during adolescence and may influence oscillatory dynamics. Second, the relatively small sample was recruited from a single regional psychiatric hospital and local schools within the same geographic area, which may introduce sampling bias and restrict demographic, socioeconomic, and cultural variability. This regional homogeneity limits the generalizability of the findings to broader adolescent populations across different contexts, and the results should therefore be interpreted as preliminary. Third, emotional states were not assessed during task performance, precluding direct evaluation of how transient affective fluctuations may influence decision-making and neural activity. Finally, while the 32-channel EEG montage employed in this study is adequate for sensor-level time-frequency analyses, its spatial resolution is limited. We therefore did not perform source localization, and our findings are interpreted at the sensor level. Future studies aiming to identify the neural generators of the observed effects would benefit from higher-density EEG arrays (≥64 channels; 94) or multimodal neuroimaging approaches. Future research should recruit larger and more diverse samples, adopt longitudinal designs to clarify developmental trajectories, employ tasks with greater ecological validity, and integrate EEG with multimodal neuroimaging approaches to better characterize the spatiotemporal dynamics of uncertainty-related decision-making and determine whether the observed electrophysiological patterns represent reliable candidate neurophysiological signatures with potential clinical or predictive utility.

## 6 Conclusion

This study provides converging behavioral and electrophysiological evidence that adolescent anxiety is associated with specific decision-making vulnerabilities and context-dependent neural dynamics that may inform future research on anxiety-related decision-making. At the behavioral level, anxious adolescents exhibited stronger avoidance tendencies during gain- and loss-related uncertain decision-making, which may be consistent with a compensatory strategy involving greater caution in uncertain contexts, thereby reducing uncertainty but limiting adaptive exploration. At the neural level, reduced β-band power during gain processing may reflect altered engagement of inhibitory and top-down control processes, while enhanced β-band connectivity between occipital and temporal regions may be associated with altered coordination of perceptual and affective-contextual processing during uncertain decision-making. Under the loss frame, stronger θ-band connectivity between parietal and temporal regions may reflect enhanced monitoring and evaluation of negative outcomes under uncertainty. These findings suggest that avoidance behavior in anxious adolescents may be associated with altered β- and θ-band dynamics involving regulatory and outcome-monitoring processes. Importantly, these interpretations are based on sensor-level EEG measures and should therefore be considered potential neurophysiological correlates rather than direct evidence of specific underlying neural mechanisms. Importantly, β- and θ-band connectivity between occipital, parietal, and temporal regions was associated with behavioral performance during uncertain decision-making, highlighting their potential relevance as candidate neurophysiological signatures of anxiety-related alterations in decision-making under uncertainty.

## Data Availability

The raw data supporting the conclusions of this article will be made available by the authors, without undue reservation.
